# Developmental Pesticide Models of the Parkinson Disease Phenotype

**DOI:** 10.1289/ehp.7570

**Published:** 2005-05-26

**Authors:** Deborah A. Cory-Slechta, Mona Thiruchelvam, Brian K. Barlow, Eric K. Richfield

**Affiliations:** 1 Environmental and Occupational Health Sciences Institute, University of Medicine and Dentistry of New Jersey and Rutgers University, Piscataway, New Jersey, USA; 2 Department of Environmental and Occupational Medicine, and; 3 Department of Pathology and Laboratory Medicine, Robert Wood Johnson Medical School, University of Medicine and Dentistry of New Jersey, Piscataway, New Jersey, USA

**Keywords:** development, dopamine, maneb, nigrostriatal system, paraquat, Parkinson disease, pesticides, substantia nigra

## Abstract

It has been hypothesized that developmental insults could contribute to Parkinson disease (PD), a neurodegenerative disorder resulting from the loss of the dopamine neurons of the nigrostriatal pathway. Two models of developmental pesticide exposures in mice are presented here that yield PD phenotypes consistent with this possibility. Combined exposures to the herbicide paraquat (PQ) and the fungicide maneb (MB), both of which adversely affect dopamine systems, administered from postnatal days 5–19, produced selective losses of dopamine and metabolites and reduced numbers of dopamine neurons in the substantia nigra. Effects were greater than those produced by adult-only exposures. Moreover, developmental PQ + MB exposures enhanced vulnerability to this pesticide regimen when administered subsequently in adulthood. In a second model, exposure to MB from gestational days 10–17 markedly increased vulnerability to PQ exposures during adulthood, with reductions in dopamine and metabolites and numbers of dopamine neurons in the substantia nigra. Females evidenced protection in both models. Collectively, these models demonstrate that developmental exposures can produce progressive, permanent, and cumulative neurotoxicity of the nigrostriatal dopamine system and enhance vulnerability to subsequent environmental insults. Finally, effects of PQ + MB were greater than those of either pesticide alone in the postnatal model. This is consistent with a multiple-hit hypothesis predicting that multiple concurrent insults occurring at different target sites within a system (here nigrostriatal dopamine) may constrict the range and flexibility of compensatory mechanisms, thereby compromising the integrity and viability of the system. As such, this hypothesis presents a biologic strategy for identifying potentially significant neurotoxic mixtures for hazard identification in future studies.

Parkinson disease (PD) is a chronic neurodegenerative condition afflicting nearly one million people in the United States alone (McDonald et al. 2003). First described almost 200 years ago (Parkinson 1817), it is characterized clinically by resting tremor, rigidity, akinesia, and bradykinesia with a subsequent loss of postural stability. The sporadic form of the disease typically begins after 60 years of age. The basis for this movement disorder is a progressive loss of dopaminergic (DA) cells in the substantia nigra pars compacta, the neurons of the nigrostriatal DA system. The consequence is a loss of DA in the nigrostriatal system and thereby its control over motor function. Symptoms become evident after the loss of approximately 80% of these DA neurons. Characteristic of the accompanying pathology is the presence of Lewy bodies, which are intracytoplasmic inclusions containing neurofilament proteins and others such as α-synuclein and parkin. PD brings with it an extensive emotional and financial toll not only on the individuals affected but also on their families and the community. The disease is primarily idiopathic or sporadic; Mendelian-based genetic mutations account for less than 5% of the total cases of PD.

The cause(s) of PD remains unknown, and over the years the mechanism of genetic inheritance versus environmental exposures has been debated. It has become increasingly clear over the past several years, however, that multiple risk factors, both genetic and environmental, can produce parkinsonism (Figure 1). No single genetic mutation can account for most PD, an assertion underscored by the findings of a study by (Tanner et al. 1999) that examined more than 19,000 pairs of white male twins from the World War II veterans registry. The results showed no difference in concordance rates for PD in monozygotic and dizygotic twins 60 or more years of age. Nevertheless, at the current time, at least nine different genetic loci have been associated with the familial form of the disease (Hardy et al. 2003). These include mutations in the genes for α-synuclein and parkin, two of the proteins found in Lewy bodies. In environmental exposures, both pesticides and metals have been implicated as risk factors for PD, based on results from some epidemiologic studies (both metals and pesticides) as well as from experimental models (pesticides). A series of meta-analyses of such studies, including 16 based on living in rural areas, 18 for well-water drinking, 11 for farming, and 14 for pesticide exposures (Priyadarshi et al. 2001), concluded that all these variables may be risk factors for the development of PD.

Consistent with the potential for environmental exposures to contribute to the etiology of PD is the fact that the disease shows geographic variation in its mortality statistics. Such variation has been reported in Japan (Imaizumi 1995), Canada (Imaizumi 1995; Svenson 1990; Svenson et al. 1993), and the United States (Kurtzke and Goldberg 1988; Lanska 1997; Lilienfeld et al. 1990; Lux and Kurtzke 1987). Three studies using U.S. data show a north-to-south gradient for age-adjusted PD mortality. Figure 2 depicts results based on 1988 U.S. National Center for Health Statistics data (Lanska 1997). The highest PD rates occurred in the Northeast, Mid-Atlantic States, the Midwest, and the Pacific Coast states, with intermediate levels in the mountain states and in the Southwest and very low rates in the South, particularly from Texas to Florida. This is in contrast to other neurologic diseases: stroke mortality rates are particularly high in the Southeastern United States (Pickel et al. 1997), and cancers of the nervous system are lowest in Pacific states including California, Oregon, and Washington (Menck et al. 1998).

Also cited as evidence in support of environmental contributions to this disease is that PD occurs in greater frequency in industrialized countries. The PD prevalence rate is reportedly much lower in China than in the United States Li et al. 1985; Tanner et al. 1987, 1989), and even in China, PD appears to be associated with industrial chemical exposures (Tanner et al. 1989). A recent study examining PD mortality and pesticide exposure in California from 1984 through 1994 reported that mortality was increased in counties using agricultural pesticides after controlling for age, gender, race, birthplace, year of death, and education (Ritz and Yu 2000). The fact that the prevalence of PD in immigrant populations is comparable to the prevalence rate in the country of destination is also indicative of an environmental exposure basis of PD. For example, the prevalence of PD in Nigeria is lower than that for U.S. African Americans (Schoenberg et al. 1988); those of African Americans and whites in the United States are reported to be similar (Schoenberg et al. 1985), even though the African populations in the United States and Nigeria are largely homogeneous genetically after controlling for age and other variables that likely differ between countries. Similarly, Americans of Japanese or Okinawan ancestry have been reported to exhibit a PD incidence similar to that of white Americans, which is higher than that in Asian countries (Morens et al. 1996).

This apparent multiplicity of risk factors supports a growing belief that PD may be multifactorial in nature rather than a disease that can be ascribed to a unitary etiology. PD may be the result of the net interactions of multiple risk factors encountered over the lifetime, that is, a lifelong bionetwork of interactions, which in addition to those promoting risk, would also include factors that have shown to be protective against PD, such as caffeine and cigarette smoking (Baron 1986; Gorell et al. 1999; Kessler and Diamond 1971; Ross and Petrovitch 2001). Such a multifactorial etiology also would be consistent with PD because the disease exhibits marked heterogeneity with respect to signs and symptoms that manifest, the age of onset, and the rate of progression. For such reasons, it may be more appropriate to think of PD not as a unitary disease entity but rather as a broader phenotype. Such a premise may also explain why early studies that examined the potential for one such pesticide, paraquat (PQ), to produce parkinsonism were not particularly compelling (Bagetta et al. 1992; Markey et al. 1986; Perry et al. 1986). Investigators became interested in PQ particularly as a pesticidal risk factor because it shares a marked structural similarity to MPP^+^ (1-methyl-4-phenylpyridinium), the most widely used experimental model of PD.

However, PQ exposure does not occur in isolation but instead occurs in conjunction with many other risk factors, including other environmental chemicals. Indeed, one can consider a type of multiple-hit hypothesis for the impact of multiple risk factors targeting the brain. Specifically, the brain may readily be able to compensate for the effects of an individual chemical alone acting on a particular system of the brain. However, when multiple target or functional sites within that particular system are attacked by different mechanisms (i.e., multiple chemical exposures or chemical exposures combined with other risk factors), the system may no longer be able to homeostatically reregulate itself, thereby leading to sustained or cumulative damage. Figure 3 provides a hypothetical example featuring a DA terminal. Four concurrent insults are portrayed. Although all four insults target the DA terminal, they do so by different mechanisms, that is, at different sites of the same system. Here, for example, insult A targets the vesicular monoamine transporter in which DA is stored; insult B attacks the enzyme converting tyrosine to DOPA (3,4-dihydroxyphenylalanine) and thus the DA metabolic pathway; insult C strikes the metabolism of DOPAC (3,4-dihydroxyphenylacetic acid) to HVA (homovanillic acid); and insult D hits the DA transporter that takes DA back up from the synaptic cleft postrelease. Multiple insults occurring concurrently at multiple sites within the system may constrict the range and flexibility of compensatory mechanisms, thereby compromising the integrity and viability of the system. As a consequence, mixtures could have effects that are more robust and more rapid in onset and that differ in character from effects produced by single exposures.

On the basis of such considerations, we posited that concurrent exposures to multiple pesticides that target the nigrostriatal DA systems but that do so through different mechanisms might provide more significant neurotoxicity (Cory-Slechta, in press). Thus, an exposure model was developed in young adult mice based on combined exposure to the herbicide PQ and the fungicide maneb (MB), based on their DA effects (Bagetta et al. 1992; Calo et al. 1990; Liou et al. 1996; Markey et al. 1986; Miller et al. 1991; Morato et al. 1989; Perry et al. 1986; Takahashi et al. 1989; Walters et al. 1999; Yoshimura et al. 1993). PQ shares a remarkable structural similarity to MPP^+^, the most widely used experimental model of the PD phenotype. MB actually enhances the effects of MPTP (1-methyl-4-phenyl-1,2,3,6-tetrahydropyridine; parent compound of MPP^+^). This model in young adult C57Bl/6 mice was produced by administration of doses of 10 mg/kg PQ, 30 mg/kg MB, or combined PQ + MB, intraperitoneally (ip) twice a week for 6 weeks, for a total of 12 doses, and was shown to result in a PD phenotype (Thiruchelvam et al. 2000a, 2000b). In this phenotype, effects of PQ + MB were found to be potentiated; that is, for some measures, effects of combined PQ + MB were found where neither pesticide administered alone had any impact. The observed effects were, moreover, highly selective for the nigrostriatal DA system and irreversible. Additionally, studies using the PQ + MB model have shown a greater vulnerability of males to the combined treatment, which is consistent with observations from epidemiologic studies of PD (Wooten et al. 2004). These studies suggest that the greater incidence of the disease reported in males in epidemiologic studies may not only be due to greater exposures to potential environmental risk factors such as pesticides but also may be related to gender-based physiologic differences. Our studies also now indicate that both aging (Thiruchelvam et al. 2003) and overexpression of mutant human α-synuclein (Thiruchelvam et al. 2004) can enhance the PD phenotype produced by PQ + MB.

## Developmental Insults and Parkinson Disease

**Figure 4:**
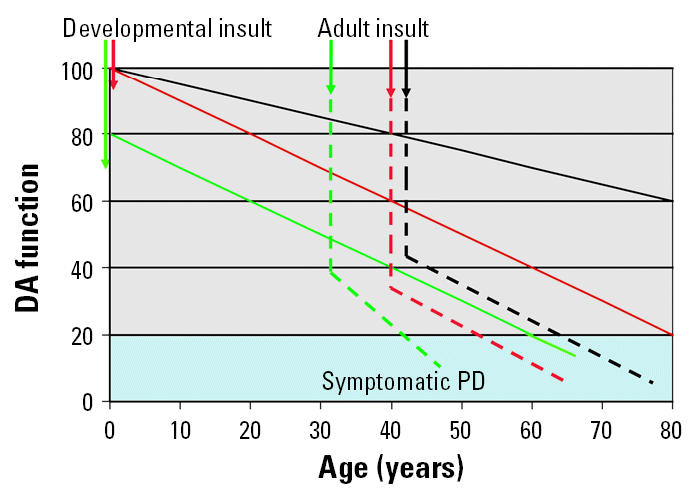
Hypothetical models by which insults incurred during development with or without added insults later in life can accelerate the onset to a PD phenotype by reaching the point where only approximately 20% of DA function remains. The solid black line depicts the rate of normal aging of the DA system; an adult insult superimposed upon this function accelerates the development of the disease (dashed black line). An insult occurring during development (solid red line) could increase the slope of the normal decline such that the level of DA dysfunction is reached earlier in life. An adult insult(s) superimposed upon this function (dashed red line) would further accelerate the process. Another model would involve a developmental insult that results in a loss of DA neurons early in life (solid green line) such that the reserve capacity is diminished and the result is a more rapid onset of the disease, again, a process that could be hastened by additional insults incurred later in life (dashed green line). Modified from Thiruchelvam et al. (2002).

Although PD is a neurodegenerative condition with a late-life onset, the possibility that it could be related to insults that occur early in life has been raised. Indeed, the pattern of manifestation of signs and symptoms should not be presumed to coincide with the timing of etiologic factors. Figure 4 depicts hypothetical models in which events occurring early in life could contribute or lead to PD later in life. Normal age-related degeneration in the DA system has been repeatedly described, which includes the loss of DA cell bodies as well as alterations that compromise the function of residual DA neurons (Anglade et al. 1997; Cabello et al. 2002; Calne and Langston 1983; Roth and Joseph 1994). Insults during the adult stage of the life cycle superimposed on such normal aging could further decrease DA function and lead more rapidly to the symptomatic PD range (< 20%; shaded area of Figure 4). It could be posited that events occurring early in development have long-term, delayed consequences for DA systems that may become evident only as the DA system undergoes further adult-related cell loss. For example, a developmental insult alone could accelerate the loss of DA neurons across life (solid red line), such that the percentage of DA function reaches the symptomatic PD range more rapidly. Adult insults superimposed upon such an enhanced decline, moreover, could further accelerate such a process (dashed red line). Alternatively, or in addition to these events, developmental insults could result in an early loss of DA neurons such that the entire curve of degeneration is displaced downward (solid green line) and again the symptomatic range is reached earlier in life, again, with such a possibility further accelerated by adult insults (dashed green line).

The potential for adverse effects of pesticide exposures to infants and children is of significant concern (Kimmel and Makris 2001; Mendola et al. 2002; Tilson 1998). Many pesticides were specifically designed to affect the nervous system of pests, but the phylogenetic parsimony of nervous system structure and function across species leaves humans at risk as well. This must be coupled with the fact that numerous factors contribute to the particular vulnerability of the developing nervous system to environmental chemical exposures. One is the complex series of events associated with brain development, which starts during the embryonic period and actually extends through adolescence. During this time, progenitor cells in the brain must travel in a defined time frame to their appropriate location and establish functional connections that are the basis for signals that ultimately underlie complex human behavior. In humans this period is primarily during the prenatal period. An additional factor operative early in life that can contribute to enhanced vulnerability of the brain is the incomplete development of the blood–brain barrier, including possible enhanced permeability to toxicants, or other examples of altered toxicokinetics. Although the barriers to blood–brain and blood–cerebrospinal fluid interfaces (i.e., tight junctions) are present from early in development, permeability to small lipid-insoluble molecules is greater in developing brain, and mechanisms of ion and amino acid transfer develop only sequentially with brain growth (Saunders et al. 1999).

Such possibilities raise at least three questions: *a*) Can developmental environmental insults lead to progressive or permanent nigrostriatal DA neurotoxicity? *b*) Will this insult alter vulnerability to subsequent environmental insults? *c*) Can such environmental insults produce cumulative neurotoxicity? The answers to such questions were pursued using the combined PQ + MB model described previously.

Subsequent studies using PQ + MB were undertaken to address the questions posed above with respect to exposures during development. Findings from these efforts attest to the significance of early development as a period of vulnerability for insults that later lead to a PD phenotype, demonstrating positive answers to all three questions. Indeed, findings from these studies also raise new and important issues concerning the adequacy of current risk assessment strategies.

## Developmental Environmental Insults: Dopamine Neurotoxicity and Altered Vulnerability

**Figure 5:**
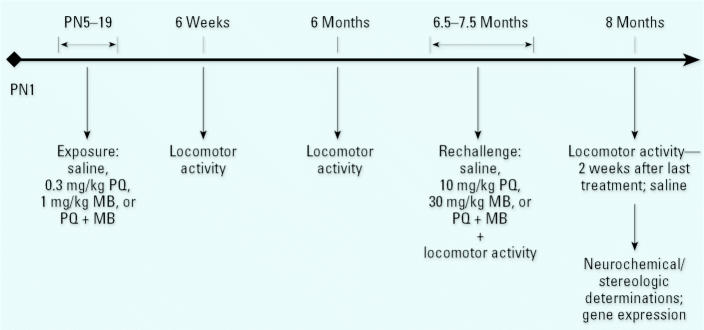
Depiction of the experimental design for the experiment examining postnatal exposure to PQ and/or MB in C57Bl/6 mice. PN, postnatal days. Pups were treated with saline, 0.3 mg/kg PQ, 1.0 mg/kg MB, or the combination administered ip from PN 5–19 of gestation. Offspring were tested for locomotor activity at 6 weeks of age and again at 6 months of age. A subset of mice were rechallenged at 6.5–7.5 months of age with saline, 10 mg/kg PQ, 30 mg/kg MB, or the combination administered twice per week for 6 weeks, for a total of 12 doses. Another set of mice were given these treatments only as adults. Approximately 2 weeks after the adult treatments, locomotor activity was evaluated and brains harvested for determinations of levels of DA and metabolites and for determinations of numbers of neurons using unbiased stereology. Modified from Thiruchelvam et al. (2002).

Can developmental environmental insults lead to progressive or permanent nigrostriatal DA neurotoxicity? Will this insult alter vulnerability to subsequent environmental insults? To address these questions related to the potential for developmental insults to lead to a PD phenotype, C57Bl/6 mice were exposed as shown in Figure 5 to the pesticides PQ + MB alone or in combination during postnatal days 5–19 at doses that were 1/30th of those used in the young adult studies (Thiruchelvam et al. 2002). Locomotor activity was assessed at 6 weeks and again at 6 months of age. In our studies, this measure has proven to be predictive of underlying nigrostriatal DA neuron loss. At 6.5–7.5 months of age, a subset of these mice were rechallenged with the same (adult) dosing regimen of the pesticides. Some mice were exposed to the pesticides only as adults. Approximately 2 weeks after the end of the adult rechallenge, locomotor activity was again evaluated, and brains were harvested thereafter for determinations of levels of DA and metabolites and for assessment of numbers of DA neurons using unbiased stereologic determinations.

**Figure 6:**
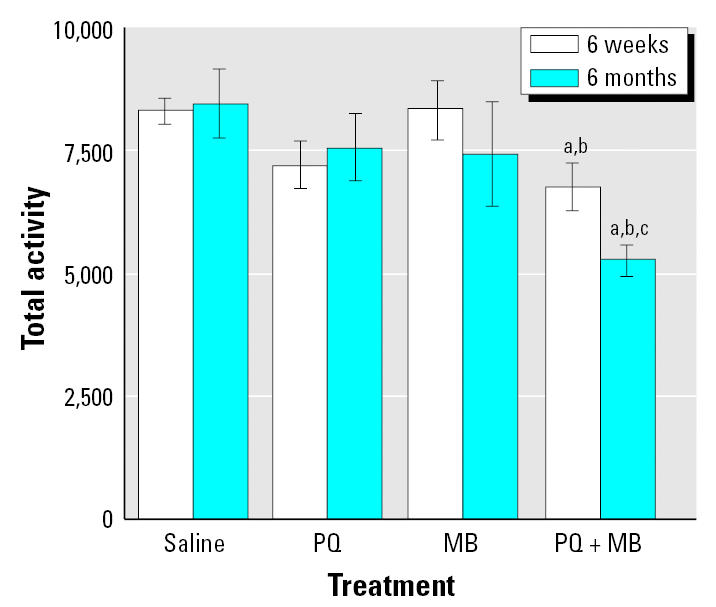
Locomotor activity changes after postnatal exposure: total locomotor activity counts in 45 min sessions measured at 6 weeks of age and again at 6 months of age in mice exposed developmentally to saline, 0.3 mg/kg PQ, 1.0 mg/kg MB, or the combination via ip administration postnatally as shown in Figure 5. Modified from Thiruchelvam et al. (2002). Significant differences were found compared with ***^a^***corresponding saline control; ***^b^***corresponding PQ group; and ***^c^***corresponding MB alone.

Figure 6 depicts total locomotor activity (total horizontal activity counts across the duration of the session) measured in 45-min sessions at 6 weeks of age and again at 6 months of age, the latter determination occurring more than 5 months after pesticide exposure. Locomotor activity reductions were seen only in the PQ + MB group and not in response to either PQ or MB alone; that is, effects were potentiated. Reductions of 23% were evident in the PQ + MB group at 6 weeks of age, and by 6 months of age levels were further reduced by 38% relative to that of controls. Thus, these effects were progressive and could also be presumed to be permanent.

**Figure 7:**
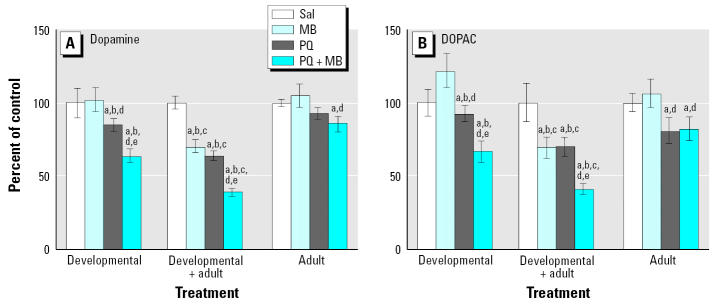
Levels of DA (*A*) and the metabolite DOPAC (*B*) measured at 2 weeks after adult rechallenge in mice exposed developmentally only to saline (Sal) or to 0.3 mg/kg PQ, 1.0 mg/kg MB, or the combination via ip administration postnatally (Developmental); developmentally followed by adult rechallenge with saline, 10 mg/kg PQ, 30 mg/kg MB, or the combination administered twice per week for 6 weeks for a total of 12 doses (Developmental + Adult); or only as adults to saline, 10 mg/kg PQ, 30 mg/kg MB, or the combination administered twice per week for 6 weeks for a total of 12 doses (Adult), as shown in Figure 5. Modified from Thiruchelvam et al. (2002). Significant differences were found compared with ***^a^***corresponding saline; ***^b^***corresponding adult only; ***^c^***corresponding developmental only; ***^d^***MB alone; ***^e^***and PQ alone.

Changes in levels of DA and its metabolites were determined in striatum at the termination of the experiment, when mice were approximately 8–9 months of age. DA and DOPAC (3,4-dihydroxyphenylalacetic acid) levels are depicted in Figure 7 for groups that were exposed only during development or only as adults and those exposed developmentally and rechallenged as adults (developmental + adult). Relative to controls, developmental exposure to PQ alone decreased levels of DA, but these reductions were notably enhanced by combined PQ + MB, whereas MB alone had no impact. Similar but far less pronounced effects were seen in mice treated only as adults, where only PQ + MB produced significant reductions. The most dramatic effects were observed in groups treated developmentally and then rechallenged as adults, where reductions occurred in response to both PQ alone and MB alone, with even more marked effects in the group receiving PQ + MB, where levels of DA were reduced by 62% relative to those of controls. Moreover, developmental exposure followed by adult rechallenge unmasked silent toxicity apparently produced by MB treatment during development: although no changes were seen in response to developmental-only MB exposure, reductions did occur when it was followed by adult rechallenge with MB. A highly similar pattern of treatment-related effects was seen for DOPAC.

**Figure 8:**
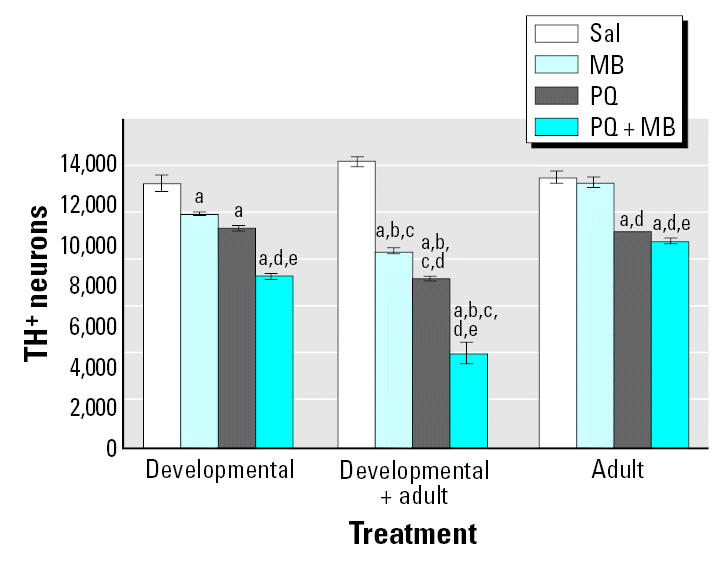
Total numbers of TH^+^ neurons in the substantia nigra pars compacta measured using unbiased stereology at 2 weeks after adult rechallenge in mice exposed as described in Figure 7. Sal, saline. Modified from Thiruchelvam et al. (2002). Significant differences were found compared with ***^a^***corresponding saline; ***^b^***corresponding adult only; ***^c^***corresponding developmental only; ***^d^***MB alone; ***^e^***PQ alone.

A similar but more marked profile of effects was observed for changes in TH^+^ neurons (Figure 8). In this case even developmental-only exposures to PQ alone and to MB alone produced modest but statistically significant decreases in numbers of DA neurons in the substantia nigra pars compacta, and even larger reductions were found for combined PQ + MB. In adult-only exposures, both PQ alone and PQ + MB produced small but significant reductions (10–15%) in numbers of TH^+^ neurons. The most dramatic effects were again seen in groups treated developmentally and then rechallenged as adults. Here, the reductions produced by PQ alone and MB alone were significantly greater than those observed in response to developmental-only exposures. So, too, was the reduction in the PQ + MB group, where the TH^+^ neuron count decreased approximately 67% relative to control. These effects were not mirrored by changes in numbers of TH^−^ neurons in the nigra (not shown) or by reductions in TH^+^ neurons in the ventral tegmental area (region of cell bodies for the mesolimbic DA system; not shown); that is, these reductions were highly selective for the nigrostriatal DA system.

**Figure 9:**
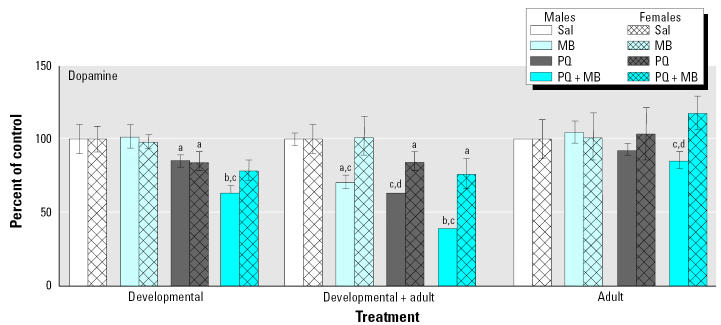
Levels of DA in male compared with female offspring measured at 2 weeks after adult rechallenge in mice exposed as described in Figure 7. Sal, saline. Male data modified from Thiruchelvam et al. (2002); female data from Thiruchelvam M, Cory-Slechta DA, Barlow BK, Richfield EK (unpublished data). Significant differences were found compared with ***^a^***saline; ***^b^***saline, PQ alone, and MB alone; ***^c^***female; ***^d^***saline and MB alone.

The protection of females from the effects of PQ + MB was particularly striking. For example, Figure 9 displays the changes in striatal levels of DA presented for males in Figure 6 and corresponding data for females. The reductions in DA levels produced by virtually all treatments were either attenuated or absent in females, with the exception of the reduction in DA in response to developmental exposure to PQ alone. The most obvious protection can be seen in comparing males with females in the groups exposed developmentally and rechallenged as adults. In these groups, females did show some reductions in levels of DA relative to those of their control group, but the magnitude of the effect was markedly reduced relative to the corresponding reductions observed in males.

**Figure 10:**
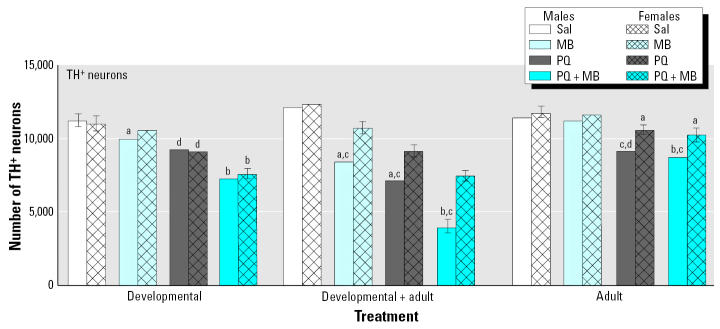
Total numbers of TH^+^ neurons in the substantia nigra pars compacta in male compared with female offspring measured using HPLC at 2 weeks after adult rechallenge in mice exposed as described in Figure 7. Sal, saline. Male data modified from Thiruchelvam et al. (2002); female data from Thiruchelvam M, Cory-Slechta DA, Barlow BK, Richfield EK (unpublished data). Significant differences were found compared with ***^a^***saline; ***^b^***saline, PQ alone, and MB alone; ***^c^***female; ***^d^***saline and MB alone.

This protection in females likewise extended to the reduction in numbers of TH^+^ neurons in the substantia nigra pars compacta produced by these pesticide exposures, as shown in Figure 10. Figure 10 shows that the reductions produced by adult exposures, and particularly by developmental exposures followed by adult rechallenge were significantly reduced in females compared with males. The reductions produced in response to developmental-only exposures were, however, of generally comparable magnitude in the two genders, suggesting a postpubertal change. Collectively, these findings showing that protection in females is conferred postpuberty suggests that events associated with maturation of reproductive systems may play a role in this response.

It is important to note that the effects described in response to these treatments were seen in the absence of any indications of systemic toxicity in treated mice, including the lack of any weight loss; indeed, mice gained weight across the experiment. Nor were any gross histopathologic changes found. Thus, the effects observed are not a reflection in any sense of a generalized toxicity.

## Can Such Environmental Insults Produce Cumulative Neurotoxicity?

**Figure 11:**
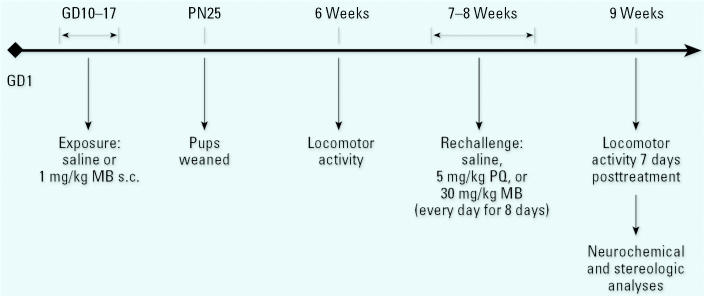
Depiction of the experimental design for the experiment examining gestational exposure to MB in C57Bl/6 mice. Abbreviations: GD, gestational days; PN, postnatal day. Dams were treated from GD 10–17 with either saline or 1.0 mg/kg MB administered sc. Offspring were tested for locomotor activity at 6 weeks of age and rechallenged at 7–8 weeks of age with saline, 5 mg/kg PQ or 15 mg/kg MB administered ip every day for 8 days. Locomotor activity was assessed 7 days after the end of the adult rechallenge, and brains were then harvested for determinations of levels of DA and metabolites and for determinations of numbers of neurons using unbiased stereology. Modified from Barlow et al. (2004).

Human exposures occur to mixtures over the life span, with the specific components of that mixture no doubt changing across time. One question raised by such exposure scenarios is whether sequential exposures across the lifetime would result in cumulative neurotoxicity to the nigrostriatal DA system. This too can be posited in the context of the multiple-hit hypothesis described previously, in that sequential and permanent damage to different target sites within the system could also result in compromise of homeostatic regulatory capacities. This question was pursued using the experimental design depicted in Figure 11. In this study C57Bl/6 mice were exposed during gestation to saline or to MB only via administration to the dam of a dose of 1 mg/kg (1/10th the dose used in young adult studies) subcutaneously (sc) during gestational days 10–17, a time frame chosen to correspond to the emergence of the nigrostriatal DA system. Pups were weaned at 25 days of age, and locomotor activity was evaluated at 6 weeks of age, again as a preliminary gauge of underlying changes in the DA system. At approximately 2 months of age, a subset of mice was challenged with saline, with 5 mg/kg PQ alone, or with 30 mg/kg MB alone, the doses used with young adult mice, and these doses were administered daily for 8 days. One week later, locomotor activity was redetermined, and brains were harvested for determinations of levels of catecholamines and stereologic assessment of numbers of DA neurons (Barlow et al. 2004).

**Figure 12:**
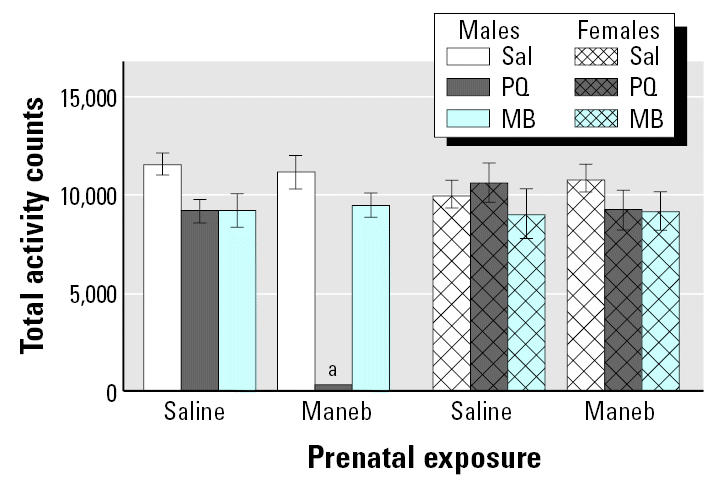
Total locomotor activity counts measured in 45 min sessions 7 days after the last adult rechallenge as described in Figure 11. Sal, saline. Values are shown for both male and female offspring. Modified from Barlow et al. (2004). ***^a^***Significantly different from all other comparisons.

**Figure 13:**
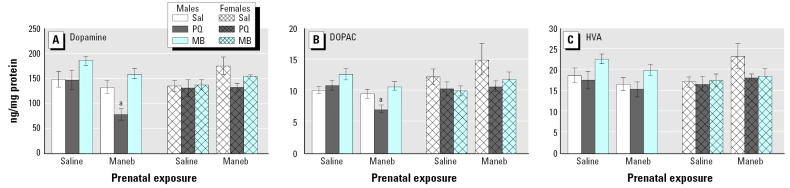
Levels of (*A*) DA and its metabolites (*B*) DOPAC and (*C*) HVA measured by HPLC 7 days after the last adult rechallenge with saline, 5 mg/kg PQ, or 15 mg/kg MB administered ip every day for 8 days after prenatal exposure to either saline or 1.0 mg/kg MB administered sc during gestational days 10–17 as shown in Figure 11. Sal, saline. Values are shown for both male and female offspring. Modified from Barlow et al. (2004) ***^a^***Significantly different from same-gender saline–saline, same-gender MB–saline, and same-gender PQ–saline.

**Figure 14:**
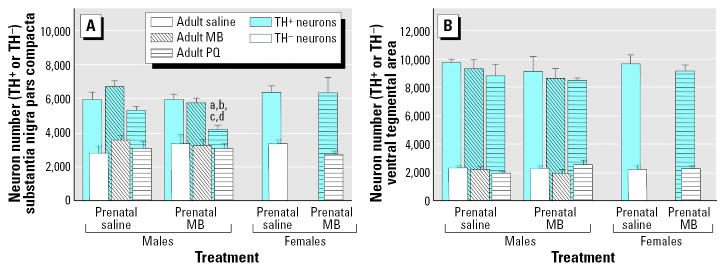
Numbers of TH^+^ and TH^−^ neurons in the substantia nigra pars compacta (*A*) and ventral striatum (*B*) measured using unbiased stereology 7 days after the last adult rechallenge with saline, 5 mg/kg PQ, or 15 mg/kg MB administered ip every day for 8 days after prenatal exposure to either saline or 1.0 mg/kg MB administered sc during gestational days 10–17 as shown in Figure 11. Values are shown for both male and female offspring. Modified from Barlow et al. (2004). Significant differences were found compared with ***^a^***same-gender Sal–Sal; ***^b^***same-gender MB–Sal; ***^c^***same-gender Sal–PQ; and ***^d^***opposite-gender same-exposure condition.

Findings from this study were largely unexpected. Changes in locomotor activity measured 7 days after the adult rechallenge are shown in Figure 12 for both males and females treated developmentally with saline or MB and rechallenged as adults with saline, PQ alone, or MB alone. With this particular exposure regimen, prenatal exposure to MB alone followed by adult rechallenge with PQ alone produced dramatic reductions in locomotor activity at this time point, effects that were seen only in males. Correspondingly, the reductions in levels of DA and of its metabolite DOPAC that were observed occurred only in response to prenatal MB exposure followed by adult rechallenge with PQ alone (Figure 13) and, again, in males but not in females. In addition, this regimen, that is, prenatal MB followed by adult rechallenge with PQ, produced a loss of TH^+^ neurons that occurred selectively in the substantia nigra pars compacta and not in the ventral tegmental area (compare Figure 13A,B). Also, there were no changes in numbers of TH^−^ neurons. Furthermore, females were protected from this loss of TH^+^ substantia nigra neurons (Figure 14) as they were from the other adverse effects of prenatal MB followed by adult PQ exposure. As in the previous study (Thiruchelvam et al. 2002), these effects were seen in the absence of any indication of systemic toxicity, body weight loss, or gross histopathology.

## Conclusions and Research Needs

These studies demonstrate the first examples of models of the PD phenotype based on developmental pesticide exposure. Our findings confirm that a developmental insult can have effects that appear to be progressive and permanent and ultimately lead to damage to the nigrostriatal DA system, including loss of TH^+^ neurons (Figures 10, 14), consistent with the hypothetical models posed in Figure 4. In addition, both the postnatal and gestational exposure experimental models examined here showed that pesticide exposures during development increased vulnerability to subsequent pesticide exposures occurring later in life. These findings indicate that vulnerability can accumulate across insults, such that the effects of successive insults may actually be enhanced. Indeed, repeated insults also revealed underlying silent toxicity, wherein effects of developmental exposures to pesticides alone manifested only after adult rechallenge with the same or even a different pesticide. These findings also underscore the need for inclusion of childhood pesticide exposures in epidemiologic studies and further evaluation in experimental models.

The basis for the protection observed in females in these studies, mainly conferred post-pubertally, is not yet clear. In addition to gender-related differences in time to onset of, magnitude of effects of and, perhaps, incidence that are reported in diseases and dysfunctions in which DA systems play a key role (e.g., attention deficit hyperactivity disorder, schizophrenia, PD), experimental studies have repeatedly demonstrated protective effects of estrogen in various experimental models, including protection against the DA toxicity associated with the experimental compound MPTP as well as against methamphetamine-induced DA toxicity (Dluzen and McDermott 2002; Dluzen et al. 1996; Miller et al. 1998). The mechanism(s) of such effects remains unknown and may be diverse but could include effects on DA release, on the DA transporter, and/or on the activity of tyrosine hydroxylase (TH), the rate-limiting enzyme in the DA synthesis pathway (Dluzen and McDermott 2000; Kuppers et al. 2000). This is a finding clearly warranting further experimental attention given the potential for therapeutic considerations.

One unexpected finding from these studies was the dramatic enhancement of the nigrostriatal dopaminergic toxicity associated with PQ treatment when it was administered in adulthood after developmental exposure to MB. The mechanisms responsible for this enhancement of toxicity, particularly given the time lag between treatments (~ 10 weeks), is not yet known. In adults the effects of PQ are potentiated by MB at least partly through a toxicokinetic interaction in which the levels of PQ in brain are increased and the rate of elimination from brain is decreased by co-administration with MB, effects that may be related to an inhibition of efflux transport of PQ by MB (Barlow et al. 2003). Clearly, such a mechanism cannot be operative in the current experiment using gestational MB followed by adult PQ given the time lag between exposures. Little is known about the neurotoxicity of MB, particularly during development. Another explanation eliminated by stereologic assessments is that the number of TH^+^ neurons is already reduced by gestational exposure to MB alone such that PQ acted to further decrease these numbers, because no decrease in TH^+^ neurons was produced by gestational MB followed by adult saline exposure (Figure 14). One possibility, however, is that MB established a “mutant steady state” (Clarke et al. 2000) in which the homeostatic state of those cells is abnormal and confers an increased rate of cell death in response to a subsequent challenge, a variant of the multiple-hit model posed in support of these experiments.

Even in the absence at the present time of an understanding of the specific mechanisms by which such augmentation of adverse effects can occur across delays of exposure between treatments, such findings raise serious questions about the adequacy of current risk assessment paradigms to encompass the patterns of toxicities observed in these studies. For example, the enhancement of the dopaminergic toxicity of PQ in adults by previous gestational exposure to MB would not necessarily have been predicted in advance; no structural similarities exist between these compounds, and they do not appear to act by identical mechanisms. Moreover, issues of cumulative toxicity, repeated insults, and exposures to mixtures are not addressed explicitly in the derivation of risk. Certainly, the question must be raised of whether the simple addition of uncertainty factors to a no-observed-adverse-effect levels in the risk assessment context would suffice to cover the toxicity produced by these sequential treatments. In addition the possibility that pharmaceuticals, herbal supplements, or food additives might interact with pesticides via toxicokinetic mechanisms, a phenomenon seen with MB potentiation of PQ effects, has not yet received sufficient consideration.

The brain, of course, consists of a complex network of highly interactive systems. This interactive matrix may account for the enhanced vulnerability of the brain under some conditions of neurotoxic chemical exposure. The occurrence of adverse effects anywhere within such circuits may result in disturbances across such networks and in associated behavioral output. Thus, the brain provides a more extensive matrix for damage than is seen in many other organs. In addition, direct effects at one point in such interactive systems may be amplified and produce indirect damage at other points within the network. Because different brain systems use common neurotransmitters for excitatory and inhibitory function, insults targeting neurotransmitter function per se, for example, receptors, transporters, or neurotransmitters, can have broad ramifications across the brain and thereby influence a wider array of behavioral functions.

The multiple-hit hypothesis of neurotoxicity upon which these studies were based postulates that insults to different target sites within a specific brain system, here the nigrostriatal DA system, when occurring concurrently or cumulatively, will compromise homeostatic and repair capacities of the system and thereby increase its vulnerability. Findings consistent with our hypothesis, as observed in these studies, suggest such a biologic plausibility rationale as a new strategy for defining potentially significant neurotoxic mixtures in a risk context for future studies, specifically mixtures of neurotoxicants acting on the same system of the brain but via different mechanisms of action.

## Figures and Tables

**Figure 1:**
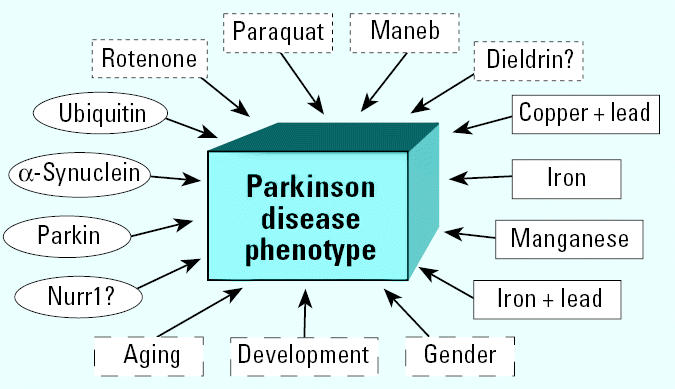
Multiple different insults lead to the PD phenotype: schematic depicting various risk factors that have been associated either with increased odds ratios for PD in epidemiologic studies or with producing characteristics of the phenotype in experimental models. Circles show genes associated with PD, rectangles depict metals implicated in PD, dotted rectangles depict pesticides implicated as risk factors, and dashed rectangles depict physiologic risk factors that contribute.

**Figure 2:**
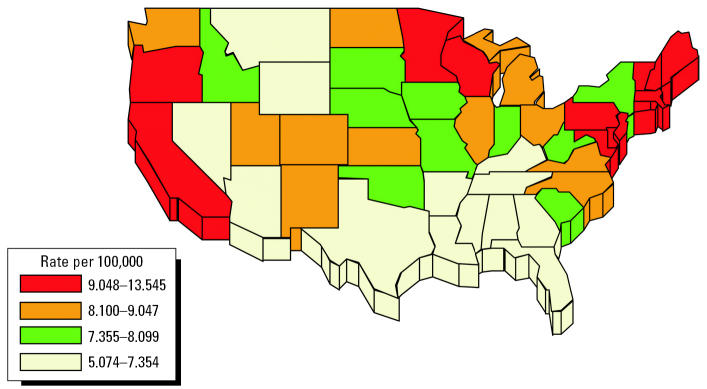
Age-adjusted mortality data for PD for states in the United States. Based on 1988 data and adapted from Lanska (1997).

**Figure 3:**
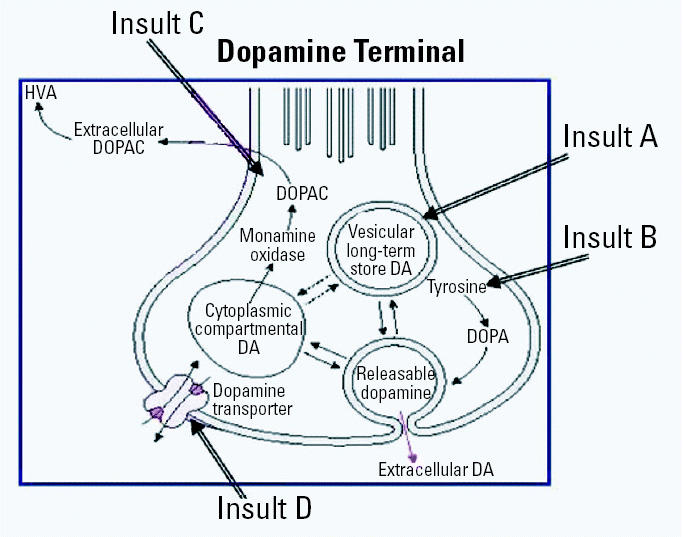
Schematic depicting the multiple-hit hypothesis as applied to a DA terminal within the central nervous system. Four concurrent insults are depicted that occur at different target sites of the DA terminal: insult A affects the vesicular transporter; insult B, affects the metabolism of tyrosine to DOPA; insult C, affects the breakdown of DOPAC; and insult D, affects the DA transporter. The multiple-hit hypothesis here presumes that the brain may readily be able to compensate for the effects of an individual chemical itself acting on a particular target system of the brain. However, when multiple target or functional sites within that particular system are attacked by different mechanisms (i.e., multiple chemical exposures or chemical exposures combined with other risk factors), the system may no longer be able to homeostatically reregulate itself, thereby leading to sustained or cumulative damage. Modified from Cory-Slechta (in press).
